# Hyperpolarized carbon 13 MRI in liver diseases: Recent advances and future opportunities

**DOI:** 10.1111/liv.15222

**Published:** 2022-03-12

**Authors:** Zheng Ye, Bin Song, Philip M. Lee, Michael A. Ohliger, Christoffer Laustsen

**Affiliations:** ^1^ Department of Radiology West China Hospital, Sichuan University Chengdu Sichuan China; ^2^ The MR Research Center, Department of Clinical Medicine Aarhus University Aarhus Denmark; ^3^ Department of Radiology and Biomedical Imaging University of California San Francisco California USA

**Keywords:** carbon 13, diffuse liver diseases, hyperpolarization, liver malignancy, magnetic resonance imaging

## Abstract

Hyperpolarized carbon‐13 magnetic resonance imaging (HP ^13^C MRI) is a recently translated metabolic imaging technique. With dissolution dynamic nuclear polarization (d‐DNP), more than 10 000‐fold signal enhancement can be readily reached, making it possible to visualize real‐time metabolism and specific substrate‐to‐metabolite conversions in the liver after injecting carbon‐13 labelled probes. Increasing evidence suggests that HP ^13^C MRI is a potential tool in detecting liver abnormalities, predicting disease progression and monitoring response treatment. In this review, we will introduce the recent progresses of HP ^13^C MRI in diffuse liver diseases and liver malignancies and discuss its future opportunities from a clinical perspective, hoping to provide a comprehensive overview of this novel technique in liver diseases and highlight its scientific and clinical potential in the field of hepatology.

**Abbreviations**
AA: acetoacetate
  ALT
  alanine transferase
  CSI
  chemical shift imaging
  CT
  computed tomography
  d‐DNP
  dissolution dynamic nuclear polarizations
  DHA
  dehydroascorbic acid
  EAA
  ethyl acetoacetate
  FDG
  fluorodeoxyglucose
  FDGal
  fluoro‐2‐deoxy‐D‐galactose
  HCC
  hepatocellular carcinoma
  HFD
  high‐fat diet
  HP
  hyperpolarized
  IRI
  ischaemia reperfusion injury
  IVIM
  intravoxel incoherent motion
  LDH
  lactate dehydrogenase
  MAFLD
  metabolic‐associated fatty liver disease
  MCD
  methionine‐choline deficient
  MRI
  magnetic resonance imaging
  NAFL
  non‐alcoholic fatty liver
  NAFLD
  non‐alcoholic fatty liver disease
  NASH
  non‐alcoholic steatohepatitis
  PC
  pyruvate carboxylase
  PDH
  pyruvate dehydrogenase
  PET
  positron emission tomography
  T2DM
  type 2 diabetes mellitus
  TAE
  transarterial embolization
  TTP
  time to peak
  VitC
  Vitamin C

**Key points**

1. Hyperpolarized ^13^C MRI is an emerging and promising technique in diffuse liver diseases and liver malignancy. It allows for real‐time assessment of hepatic metabolism *in vivo* and has been applied in detecting, monitoring progression and assessing treatment efficacy of liver diseases.
2. Great efforts should be made to optimize the workflow of hyperpolarized ^13^C MRI and foster its clinical translations, which also introduces future opportunities in this field.

## 1 INTRODUCTION

The liver plays an important role in blood filtration, substance storage and all metabolic processes in the body, especially in the regulation of glycolysis and gluconeogenesis.^1^ Aberrated metabolism is often directly linked to various liver pathological conditions.^2^ It is worth noting that chronic diffuse liver diseases, such as non‐alcoholic fatty liver disease (NAFLD) and cirrhosis, are becoming global health concerns and require early diagnosis and proper management.^3^ Likewise, liver cancer is the fourth leading cause of cancer‐related death in the world^4^, ^5^ and imposes significant burdens on healthcare systems.

At present, histopathological assessment of hepatic tissues obtained by biopsy or resection remains the gold standard in most liver diseases. However, this invasive approach is prone to sampling error and may cause complications,^6^ which is less suitable for longitudinal studies, therefore requiring non‐invasive tools. Medical imaging has been widely applied in the detection, diagnosis and monitoring of liver diseases, including computed tomography (CT), magnetic resonance imaging (MRI) and positron emission tomography (PET). Images from CT and conventional proton MRI mainly reflect structural and functional changes and cannot assess altered metabolism. Non‐invasive monitoring of metabolic changes may provide insight into understanding impaired or cancerous hepatic tissues. Currently, PET is the only imaging modality that provides metabolic information in the clinical setting. It enables visualization of hepatic glucose uptake by injecting ^18^F labelled fluorodeoxyglucose (FDG) or fluoro‐2‐deoxy‐D‐galactose (FDGal) but fails to capture downstream metabolism that is often crucial in many pathological circumstances.^7^ Hyperpolarized (HP) MRI with carbon‐13 (^13^C) labelled substates, also known as HP ^13^C MRI, can uniquely track real‐time metabolic uptake and conversion *in vivo*, which would be valuable in liver diseases.^8^

With the invention of dissolution dynamic nuclear polarization (d‐DNP), dramatically enhanced signal of HP ^13^C MRI can be detected within seconds and up to minutes after injection.^9^ By mapping the dynamic conversion between HP ^13^C substrates and their metabolic products, for example, [1‐^13^C]pyruvate to [1‐^13^C]lactate or [1‐^13^C]alanine, researchers can obtain additional data beyond routine imaging modalities and form a better understanding of liver pathogenesis. Recent evidence indicates that this technique holds great promise in both diffuse liver diseases^10^, ^11^, ^12^ and liver malignancies,^13^, ^14^, ^15^ with special focus on detection and diagnosis; assessment of disease progression and prediction of therapeutic responses. Therefore, this paper aims to provide a comprehensive overview of HP ^13^C MRI in liver diseases by introducing the workflow of this technique and then reviewing its recent applications in diffuse liver diseases and liver malignancies. More importantly, we will discuss the future opportunities in this field from a clinical perspective, hoping to highlight the scientific potential of this novel technique and foster its clinical translations in hepatology.

## 2 HEPATIC METABOLISM AND PYRUVATE

The liver is an essential metabolic organ in the body, responsible for the metabolism of carbohydrates, lipids and proteins, with pyruvate serving as the key branch point of multiple metabolic pathways. As a highly biologically relevant probe, HP [1‐^13^C]pyruvate is rapidly taken up into hepatocytes following intravenous injection and metabolized into either [1‐^13^C]lactate via lactate dehydrogenase (LDH) or [1‐^13^C]alanine via alanine aminotransferase (ALT) in the cytoplasm. HP [1‐^13^C]pyruvate can also be transported into the mitochondria and converted by pyruvate dehydrogenase (PDH) into acetyl‐coenzyme A and [^13^C]CO_2_, which is in rapid equilibrium with [^13^C]bicarbonate. Based on the different resonance frequencies (i.e. chemical shift) of the substrate and products, the signals of injected pyruvate and its metabolites can be detected on HP ^13^C MRI.

**FIGURE 1:**
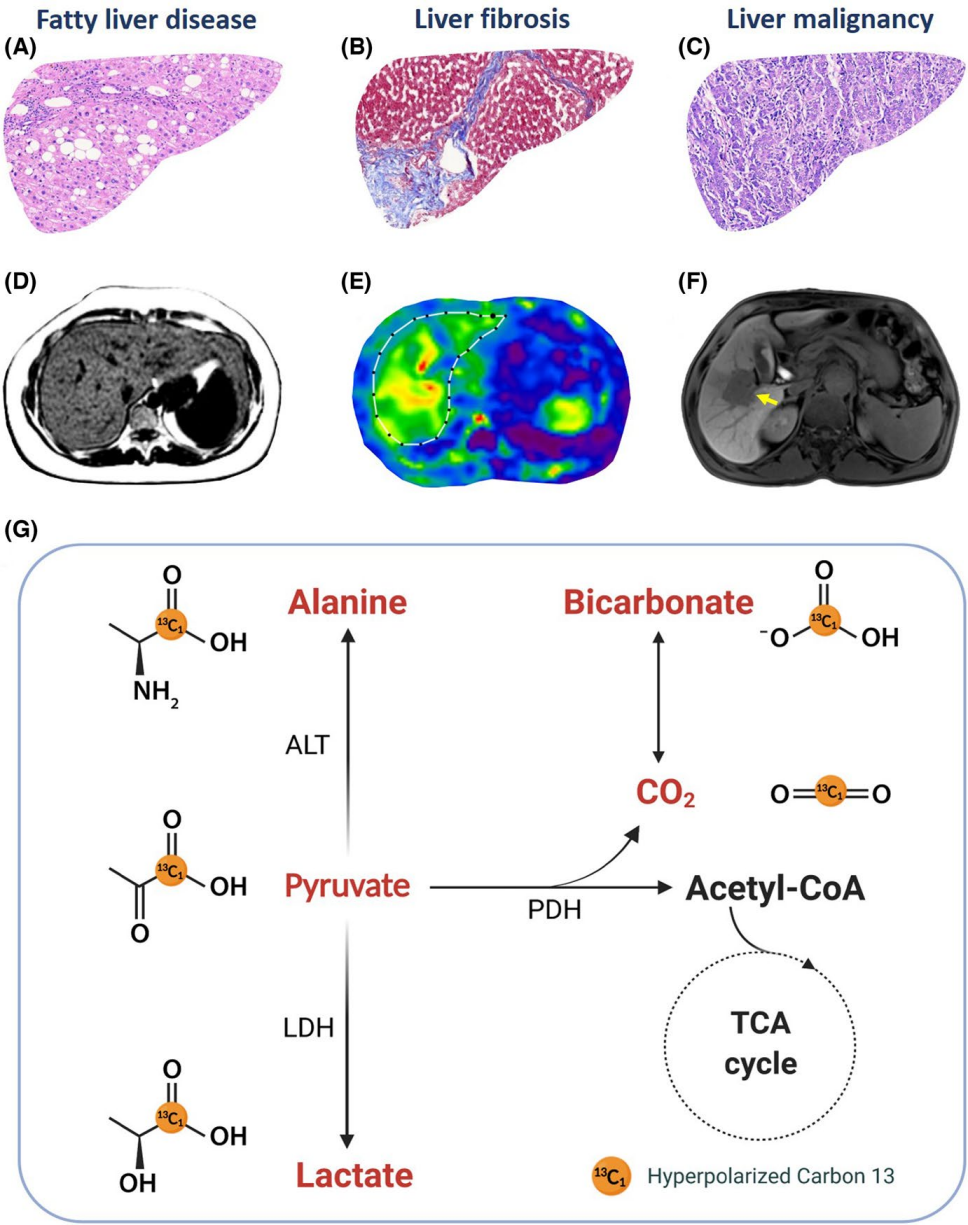
The histological changes, representative conventional images and typical metabolic pathways of different liver diseases. Fatty liver disease usually demonstrates accumulated fat droplets in histology (A) and high proton density fat fraction (D). Liver fibrosis is characterized by excessive collagen deposition in Masson’s trichrome staining (B) with increased stiffness in magnetic resonance elastography (E). The liver malignancy typically manifests as poorly differentiated malignant cells (C) and a hypointense lesion (yellow arrow) in hepatobiliary phase (F). These liver diseases closely associate with the change of metabolic activity (G). ALT, alanine transferase; LDH, lactate dehydrogenase; PDH, pyruvate dehydrogenase; TCA, tricarboxylic acid

Hepatic metabolism is tightly controlled by a variety of factors and it changes under different liver pathological conditions.^16^ HP [1‐^13^C]pyruvate is to date the only probe available for human examinations and thus widely studied. It has been predominantly used to interrogate metabolism associated with early and precise liver disease diagnosis and disease progression evaluation. For example, the activity of ALT is usually elevated in patients with liver injury, which can lead to the increase of [1‐^13^C]alanine. Abnormal glucose and lipid metabolism also often occurs in NAFLD patients.^17^ In addition, metabolic reprogramming in hepatocellular carcinoma (HCC), especially the increased lactate production,^18^ can be identified by HP ^13^C MRI. Compared to current imaging methods, HP ^13^C MRI provides unique metabolic information about pathway‐specific alterations without using ionizing radiation. This additional metabolic information opens up possible novel diagnostic and therapeutic opportunities; therefore, the multiparametric MRI examination with ^1^H and ^13^C imaging will likely be the future direction for the comprehensive assessment of liver diseases (Figure 1).

## 3 WORKFLOW OF HYPERPOLARIZED CARBON 13 MRI

**FIGURE 2:**
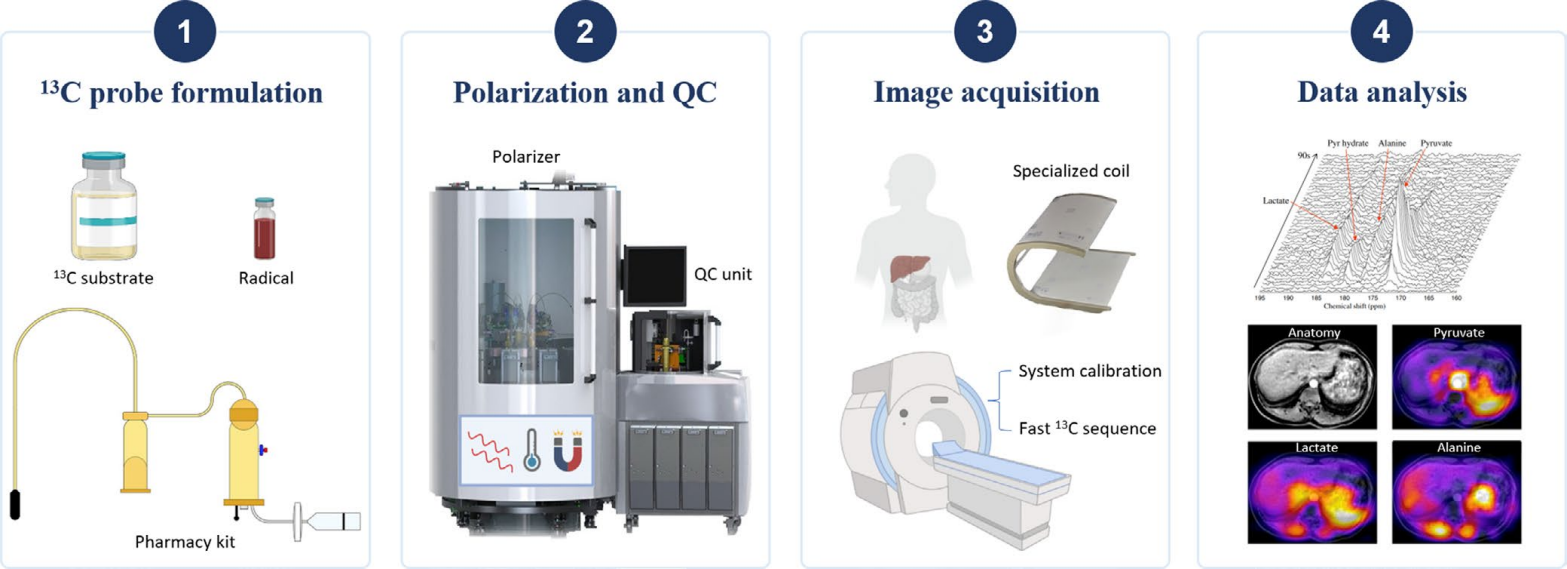
The workflow of hyperpolarized carbon 13 MRI in liver diseases. QC, quality control. The illustrations of pharmacy kit and ^13^C spectra were reproduced with permission from reference 63 and reference 25

The workflow of HP ^13^C MRI is summarized in Figure 2 and can be simply divided into following four steps. The first step is to formulate the ^13^C‐enriched compounds by mixing ^13^C substrate, typically [1‐^13^C]pyruvate and a radical with unpaired electrons. This preparation process should be conducted in a sterile environment and with the help of commercially available pharmacy kit if it is a human clinical study. Following this procedure, the sample is then placed into the polarizer to achieve hyperpolarization, where the solution experiences microwaves, cold temperature (0.8 K) and strong magnetic field (5T). Based on the d‐DNP technique, the unpaired electron spins can be polarized to nearly 100% in such environment, and the high polarization can be transferred to the ^13^C isotope in ^13^C‐enriched pyruvate molecule. Current clinical polarizers can reach up to 50% polarization of [1‐^13^C]pyruvate (MR signal is enhanced >10 000 times),^19^ which takes approximately 120 min. After the sample is polarized, a quality‐control module measures its polarization level, pH, temperature, radical and pyruvate concentrations before injection to ensure it is safe for clinical use. As for the image acquisition, specialized ^13^C radiofrequency coils are used for imaging the ^13^C hyperpolarized molecule signals, preferably dual‐tuned ^1^H/^13^C coils, allowing both anatomical and hyperpolarized ^13^C imaging in a clinical workflow. Prior to injection, the scanner is calibrated for carbon imaging, including careful shimming of the main magnetic field and adjustment of the radiofrequency centre frequency. Following the injection of the HP ^13^C substrate, the subject is imaged with optimized fast ^13^C sequences (typically less than 2 min), which would acquire the uptake and subsequent metabolic conversion in the liver parenchyma. In order to evaluate focal disease, imaging methods, such as chemical shift imaging (CSI) and spectral spatial excitation imaging, are the preferred option for liver applications. Finally, with the post‐processing algorithms, the acquired data can be quantified and visualized for the exploration of metabolic alterations.^20^ One common method is to express the total signal of downstream metabolites as a fraction of the signal from the injected probe. Alternatively, quantitative metabolic measures such as the apparent rate constant for pyruvate‐to‐lactate conversion (k_PL_) can also be calculated and quantify changes in specific drivers of the glycolysis.

Current technical challenges in HP ^13^C MRI include the non‐renewable HP ^13^C signal, limited spatial resolution as well as unstandardized imaging procedures. The complexity and high expense of this technique may also limit its role as a primary screening and diagnostic tool in clinical practice.

## 4 RECENT ADVANCES

A summary of recent HP ^13^C MRI studies on diffuse and focal liver diseases is shown in Table 1 and Table 2. Hereinafter, we will review the results and describe the possible clinical applications of HP ^13^C MRI in liver injury, liver fibrosis and fatty liver diseases, as well as liver malignancies.

**TABLE 1:** Summary of HP ^13^C MRI in diffuse liver diseases

Authors	Year	Probes	Subjects	Possible clinical applications
Lee et al^12^	2013	[1‐^13^C] pyruvate	Mice with high‐fat diet induced diabetes	Assessment of drug response
Josan et al^24^	2015	[1‐^13^C] pyruvate	Rats with CCl_4_ induced liver inflammation	Detection of liver injury
Kim et al^25^	2016	[1‐^13^C] pyruvate	Rats with 1,3‐DCP induced hepatotoxicity	Detection of liver injury
Kim et al^34^	2016	[1‐^13^C] pyruvate	Rats with high‐fat diet induced obesity	Detection of liver steatosis
Moon et al^35^	2017	[1‐^13^C] pyruvate	Rats with high‐fat diet induced NAFLD	Longitudinal assessment of NAFLD
Wilson et al^40^	2017	[1‐^13^C] DHA	Mice with MCD diet induced NASH	Detection and monitoring of NASH
Moon et al^29^	2018	[1‐^13^C] pyruvate	Rats with surgery induced hepatic IRI	Evaluation of liver injury
Moon et al^10^	2019	[1‐^13^C] pyruvate	Mice with TAA induced liver fibrosis	Detection and staging of liver fibrosis
Smith et al^11^	2021	[1‐^13^C] pyruvate	Pigs with western diet induced NAFLD	Detection and monitoring of NAFLD

Abbreviations: 1,3‐DCP, 1,3‐dichloro‐2‐propanol; CCl_4_, carbon tetrachloride; DHA, dehydroascorbic acid; IRI, ischaemia reperfusion injury; MCD, methionine‐choline deficient; NAFLD, non‐alcoholic fatty liver disease; NASH, steatohepatitis; TAA, thioacetamide.

**TABLE 2:** Summary of HP ^13^C MRI in liver malignancies

Authors	Year	Probes	Subjects	Possible clinical applications
Gallagher et al^58^	2008	[5‐^13^C] glutamine	HepG2	Assessment of tumour proliferation
Yen et al^50^	2010	[1‐^13^C] pyruvate	Rats with Morris hepatoma	Detection of early HCC
Hu et al^46^	2011	[1‐^13^C] pyruvate	Mice with *Myc* gene‐driven liver cancer	Detection of early HCC
Darpolor et al^49^	2011	[1‐^13^C] pyruvate	Rats with Morris hepatoma	Detection of early HCC
Von Morze et al^59^	2011	[^13^C] urea	Tumour‐bearing mice from a transgenic model	Assessment of tumour perfusion
Menzel et al^48^	2013	[1‐^13^C] pyruvate	Rats with Morris hepatoma	Combination with PET
Cabella et al^67^	2013	[5‐^13^C] glutamine	McA‐RH7777, rats with Morris hepatoma	Evaluation of drug response
Jensen et al^51^	2015	[1,3‐^13^C_2_] EAA	HepG2, McA‐RH7777, rats with Morris hepatoma	Detection of early HCC
Düwel et al^14^	2016	[^13^C,^15^N_2_] urea, [1‐^13^C] pyruvate, [1,4‐^13^C_2_] fumarate	Rats with Morris hepatoma	Evaluation of TAE response
Perkons et al^13^	2020	[1‐^13^C] pyruvate	HepG2, HR2, rats with DEN‐induced HCC	Evaluation of TAE response
Chen et al^15^	2020	[1‐^13^C] pyruvate	Patients with metastatic liver cancer	Evaluation of drug response
Bliemsrieder et al^56^	2021	[1‐^13^C] pyruvate	Rats with DEN‐induced HCC	Assessment of HCC phenotypes

*Note*: Morris hepatoma model was built by injecting McA‐RH7777 cells in rats; HR2 indicated the tumour cells derived from DEN‐induced HCC rats.

Abbreviations: DEN, diethylnitrosamine; EAA, ethyl acetoacetate; HCC, hepatocellular carcinoma; HepG2, human hepatoma cells; PET, positron emission tomography; TAE, trans‐arterial embolization.

### 4.1 Diffuse liver diseases

#### 4.1.1 Liver injury and liver fibrosis

Liver injury is commonly induced by excess alcohol consumption, drug intake and virus infection, which may progress to liver failure without timely intervention and thus requires early detection.^21^, ^22^, ^23^ Most liver injuries are initiated by disturbances in cellular metabolism and present as a group of damaged hepatocytes in histology. As revealed by recent studies,^24^, ^25^ the changed metabolism of liver impairment can be identified by HP ^13^C MRI. By intraperitoneally injecting CCl_4_ in rats, Josan et al.^24^ established an inflammatory liver injury model and subsequently acquired the dynamic metabolic maps on them. They found both alanine/pyruvate and lactate/pyruvate ratios were higher in CCl_4_‐treated group compared with control group and noted elevated activity of the ALT and LDH respectively. Such ability of HP ^13^C MRI greatly supplements the deficiency of routine clinical blood tests for measuring ALT and LDH, as it can observe the actual changes of corresponding enzymatic activities and its spatial distribution. Similarly, Kim et al.^25^ also suggested the levels of lactate and alanine as biomarkers in a preclinical hepatotoxicity model with administration of 1,3‐dichloro‐2‐propanol. In animals administered the toxic chemical, liver damage was shown histologically, including haemorrhage in hepatic parenchyma, inflammatory cell infiltration, vacuolation as well as degeneration and necrosis of hepatocytes. More importantly, a significant increase of the lactate to total carbon ratio and alanine to total carbon ratio was reported. These works confirmed the feasibility of HP ^13^C MRI in detecting liver injury, which indicates strong potential for clinical translation as it may help to monitor the disease progression and provide opportunity for treatments.

In addition, liver damage can be caused by ischaemia reperfusion injury (IRI). Typically, vascular clamping is often required in major liver resection or liver transplantation to avoid excessive bleeding, which can induce a period of hepatic ischaemia. When the blood flow is restored, the reperfusion could worsen cell injury on the already ischaemic liver and precipitate tissue necrosis.^26^ This phenomenon is called hepatic IRI and can lead to acute liver failure.^27^ Generally, alterations in biochemical and histopathological characteristics along with elevated levels of transaminases would occur during hepatic IRI, reflecting hepatocyte injury or stress.^28^ With the application of HP ^13^C MRI, the additional metabolic changes of hepatic IRI can be captured in real time and *in vivo*. A recent study from Moon et al.^29^ showed that IRI rats had increased alanine and lactate but decreased pyruvate levels, compared with both sham‐operated controls and rats before IRI. They considered higher alanine level as an indicator of increased vulnerability of hepatic function to IRI and associated the higher lactate level with increased LDH activity. Taken together with previous findings, HP ^13^C MRI and its metabolic biomarkers provide better understanding of mechanism related to liver injury and show great promise in diagnosing and monitoring liver injury.

Repeated wound‐healing response of liver injury can lead to accumulation of extracellular matrix and cause liver fibrosis, which progressively restricts normal hepatic regeneration, thereby increasing the risk of hepatic dysfunction, portal hypertension and even HCC.^30^ Early or intermediate liver fibrosis can be reversed with elimination of causative injury triggers and the application of anti‐fibrotic drugs,^31^ introducing the urgent need for its early detection and accurate staging in a non‐invasive manner. Recently, Moon et al. applied HP ^13^C MRI in a liver fibrosis animal model induced by thioacetamide.^10^ Their findings demonstrated elevated levels of lactate and alanine in fibrosis groups compared to normal control group, validating the potential alterations of glycolysis and gluconeogenesis in hepatic fibrogenesis, and also suggested the ratios of alanine/pyruvate and alanine/total carbon as the indicators in assessing the severity of liver fibrosis. Moreover, negative correlation was presented between the level of HP ^13^C metabolites and pseudo‐diffusion coefficient (*D* ^*^) derived from intravoxel incoherent motion (IVIM), which reflected perfusion‐related information and was widely used to evaluate liver fibrosis.^32^ Hence, in conjunction with functional imaging parameters, one could expect the future role of HP ^13^C MRI in detecting and staging liver fibrosis and aiding appropriate clinical therapeutic decisions.

#### 4.1.2 Fatty liver diseases

NAFLD is characterized by excessive accumulation of fat in the liver and is histologically classified as non‐alcoholic fatty liver (NAFL) and non‐alcoholic steatohepatitis (NASH), with the latter presenting hepatic inflammation and oxidative stress.^33^ Owing to the dramatic changes in lifestyles and diet, the prevalence of NAFLD has increased in the past decades and has posed serious health and economic burdens worldwide.^3^ Accurate stratification of liver steatosis and early identification of NAFLD are of utmost importance in disease management, making the novel non‐invasive imaging technique, HP ^13^C MRI, a promising tool in this context. Consecutive studies from Jeong’s group initially explored the time course changes of HP ^13^C metabolites in obese and NAFLD rats induced by high‐fat diet (HFD), suggesting the increased levels of alanine and lactate as the useful biomarkers of fatty liver diseases.^34^, ^35^ Subsequently, Smith et al. quantified the metabolic changes in NAFLD and normal pigs by measuring time to peak (TTP) of HP ^13^C pyruvate and its metabolites.^11^ They noted the decreased liver lactate TTP in ^13^C spectra in NAFLD pigs, indicating an increased rate of lactate production and a disturbance in liver lipid synthesis. Based on these findings, it is anticipated to incorporate HP ^13^C MRI in clinical practice to properly understand the underlying mechanisms of NAFLD and eventually guide clinicians to better manage this disease and help its reversal.

A growing body of evidence suggests that NAFLD is greatly associated with metabolic dysfunctions, such as insulin resistance and type 2 diabetes mellitus (T2DM)^36^; therefore, the nomenclature was recently revised to metabolic associated fatty liver disease (MAFLD),^37^ allowing for a wider definition of the disease. A pioneer study by Lee et al. successfully utilized HP ^13^C MRI to determine the changes in hepatic gluconeogenesis in HFD‐induced mouse model of T2DM.^12^ Particularly, compared with control group, increased exchange rates of pyruvate to aspartate and malate were demonstrated in HFD mice, with the former exchange rate exhibiting significant correlation with gluconeogenic pyruvate carboxylase (PC) activity, suggesting the critical role of the PC pathway in hepatic glucose production. What is more, they showed that HFD mice treated with metformin displayed lower aspartate and malate signals as well as decreased exchange rates from pyruvate, agreeing with downregulated gluconeogenesis. Encouraged by the capability of HP ^13^C MRI in probing metabolism that were previously inaccessible in other imaging modalities, we believe this non‐invasive technique may facilitate identification of novel therapeutic targets and longitudinal assessment of therapeutic response in MAFLD patients.

Given the adverse consequences of NASH, namely liver cirrhosis and cancers, it is crucial to recognize the disease at an early stage and initiate corresponding medical treatments. The ongoing research for pharmacological treatment and therapeutic targets necessitates an understanding of the metabolic pathways in this field.^38^ Fortunately, HP ^13^C MRI offers additional ^13^C probes alternative to pyruvate, such as [1–^13^C]dehydroascorbic acid (DHA), which can be converted to Vitamin C (VitC) in the liver and used for exploring redox reaction *in vivo*.^39^ Wilson et al. induced a NASH animal model with methionine‐choline deficient (MCD) diet and then intravenously injected HP ^13^C labelled DHA in them.^40^ In relative to control group, a 49% reduction in the ratio of DHA to VitC was observed in mice with MCD diet, accompanied by hepatic fat deposition. Notably, the alterations in metabolic ratios returned to baseline when placing the previous MCD animals on a normal diet for 1 week. Even though the exact metabolic and catalytic process can be difficult to identify under the complexity of the intracellular redox network, there is no doubt that HP ^13^C MRI has broadened the current knowledge of NASH‐related metabolic abnormalities, which could possibly introduce novel therapeutic chances in near future.

### 4.2 Liver malignancy

#### 4.2.1 Detection of early HCC

Typical hepatocarcinogenesis is considered as a stepwise development, from regenerative nodules, to dysplastic nodules and early HCC, and finally to overt HCC.^41^ This multistep progression is accompanied by morphological, histopathological and haemodynamic changes, as well as altered metabolism.^42^, ^43^, ^44^ However, since a majority of HCCs arise in the cirrhotic background, which consists of a heterogeneous structure and displays as multiple mass‐like nodules,^45^ the detection of small or early HCC can be extremely challenging with conventional imaging modalities, making metabolic imaging a potential tool in this regard. By investigating an animal model of *Myc* gene‐driven liver cancer with HP ^13^C MRI, Hu et al. found that tumour metabolic alterations preceded any observable morphological and histological changes.^46^ More specifically, their findings showed a significant increase in the conversion of pyruvate to alanine in pretumor tissues, which was absent in either normal tissues or established tumours, and they also indicated the precancerous regions with the most abundant alanine signal tended to eventually develop into tumours. In clinical scenarios, most HCC patients are diagnosed at an advanced stage and have limited treatment options; therefore, these promising results of HP ^13^C MRI in HCC early detection are of great importance, which provides the possibility of capturing early tumorigenesis and identifying it before tumour formation.

Consistent with prior knowledge of the Warburg effect that cancer cells prefer glycolysis rather than oxidative metabolism despite the adequate oxygen supply,^47^ HCC is characterized by increased flux of pyruvate to lactate, which can be readily detected by HP ^13^C MRI.^46^, ^48^ Moreover, significant alanine production was also noted in HCC cells and animal model with implanted Morris hepatoma in a study from Darpolor et al.^49^ They reported increased lactate and alanine in tumour tissues and attributed these changes to elevated LDH and ALT activity. Interestingly, the conversion from pyruvate to alanine significantly superseded that of pyruvate to lactate in their study, which was in good agreement with a previous literature with same animal model.^50^ However, controversial results were demonstrated in studies with different animal models^46^ or different tumour‐implanted locations,^48^ showing higher lactate signal than alanine in tumour tissues, which may have been owing to the difference in tumour growth microenvironment and vascularization. Thus, although these metabolic signatures from HP ^13^C MRI may help to unveil the underlying mechanisms of HCC development and facilitate tumour detection and differential diagnosis, metabolic aberrations in patients with primary liver malignancy remain unknown and require extended investigations in the future.

More recently, new HP ^13^C probe, [1,3‐^13^C_2_]ethyl acetoacetate (EAA), has been reported feasible in imaging the metabolism in rats with implanted HCC.^51^ It is generally acknowledged that the concentrations and activities of carboxylesterases, an enzyme that turn EAA to acetoacetate (AA), are lower in cancer cells compared to the corresponding normal cells.^52^ In particular, as shown by Jensen et al,^51^ approximately four times higher substrate‐to‐product signal ratio was observed in tumour tissues when compared to the surrounding healthy tissue. Furthermore, in comparison with the images from commonly used pyruvate, the contrast to noise ratio of images was significantly improved by using EAA. These findings of new metabolic biomarkers in HP ^13^C MRI are promising because they offer an opportunity to develop novel strategies for enhancing image contrast between cancerous and normal tissues and ultimately improving the detection of small and early cancer lesions.

#### 4.2.2 Assessment of biological characteristics

Although most HCCs have similar imaging features, different biological characteristics do exist in HCCs, which can significantly affect treatment efficacy and prognosis.^53^ As pyruvate and its products showed great potential in identifying tumour aggressiveness in several tumour entities,^54^, ^55^ the various biological characteristics in HCCs could be noninvasively evaluated with the help of HP ^13^C MRI. In order to better delineate the metabolic phenotyping of HCCs and explore its correlation with tumour biological behaviours, Bliemsrieder et al. recently conducted a sophisticated study, firstly inducing endogenous HCC in rats, and then re‐implanting the extracted tumour cells in another group of nude rats.^56^ Their findings showed different lactate‐to‐alanine signal ratios of endogenous HCCs and higher lactate signal in re‐implanted tumours derived from high lactate‐to‐alanine ratios tumour cells. Additionally, they suggested that HCCs with high lactate‐to‐alanine ratios may be more aggressive because high lactate production was reported to be associated with higher biological aggressiveness in cancers.^57^ In other words, metabolic alterations detected by HP ^13^C MRI, especially glucose metabolism and lactate production, provide more insights into biological characteristics of liver malignancy and therefore may promote the development of additional diagnostic and prognostic biomarkers.

Alternative HP ^13^C probes were also introduced in the evaluation of tumour biological characteristics.^58^, ^59^ Previous evidence suggested that HCCs with highly proliferated properties tend to be more aggressive,^60^ leading to early recurrence and poor prognosis, which makes [5‐^13^C_1_] glutamine a promising substrate in HP ^13^C MRI. Physiologically, glutamine can be converted to glutamate by intramitochondrial glutaminase. It plays an essential role in tumour cell metabolism and is related to cell proliferation.^61^ An initial study confirmed the feasibility of HP ^13^C MRI in imaging the conversion from glutamine to glutamate in human hepatoma cells,^58^ which may allow for the assessment of tumour proliferation and the prediction of prognosis in patients with HCC in the future. Apart from metabolically active HP ^13^C substrates, inactive agents, such as [^13^C]urea, can be possibly used for assessing biological characteristics in liver malignancy with its promising results in perfusion imaging.^62^, ^63^ Von Morze et al. found significant differences in regional perfusion characteristics in cancerous tissues by injecting HP [^13^C] urea in a preclinical HCC model.^59^ In particular, a 19% reduction in mean blood flow was observed in tumours, whereas 26% elevation was found in the tumour rim. These results and the described method were clinically relevant because tumours typically exhibit altered blood flow and perfusion patterns owing to abnormal neovascularization. Therefore, with the obtained perfusion information by HP ^13^C MRI, researchers could probably predict the biological behaviours of HCC and patients may finally benefit from individual pre‐therapeutic tumour assessment.

#### 4.2.3 Evaluation of treatment response

Unresectable HCCs are usually recommended for locoregional treatment, including transarterial embolization (TAE) with or without chemo drugs, or systemic therapy.^64^ Nevertheless, frequent local recurrence and metastasis after treatment can be problematic in refractory HCCs, which results in poor overall survival and high mortality in patients.^4^ Current imaging paradigms mostly provide information on tumour volume and vascularity for the assessment of treatment efficacy,^65^ which fails to detect the latent tumour cells and emphasizes the need for advanced metabolic imaging methods, such as HP ^13^C MRI. Recently, Perkons and his colleagues found that latent HCC cells activated metabolic reprogramming to survive TAE‐induced ischaemia.^13^ Notably, decreased anabolism and increased lactate production were observed in latent HCC undergoing TAE‐induced ischaemia both *in vitro* and *in vivo*. In addition, as illustrated in the representative images in their study, conventional proton imaging failed to detect viable tumour cells with absent contrast enhancement, while HP ^13^C MRI enabled the direct detection of persistent metabolism in surviving HCC cells at the tumour periphery. Together, these inspiring findings hold significant implications for evaluating therapeutic response and guiding subsequent interventions in patients with unresectable HCC and help to improve their clinical outcomes.

Pathologically, necrosis is usually the consequence of embolizing feeding hepatic arteries and depriving tumours from nutrients in TAE treatment, which can be easily detected by using another HP ^13^C probe, [1,4‐^13^C_2_]fumarate. In general, the conversion rate and concentration of fumarate and its metabolite (i.e., malate) would rapidly change when the tumour cells are in a necrotic condition without intact membrane.^66^ To simultaneously collect the information about tumour metabolism, perfusion and necrosis after TAE therapy, Düwel et al. performed a HP ^13^C MRI experiment with multiple HP substrates, including pyruvate, urea and fumarate, in a rat model with orthotopic HCC.^14^ As expected on TAE treatment, decreased urea and pyruvate signals, as well as an increased apparent conversion rate from pyruvate to lactate were observed in their study, indicating reduced perfusion and increased hypoxic glycolysis. Furthermore, there was a significant elevation of malate after embolization and the ratio of total malate to total fumarate greatly correlated with the histological necrosis level, with correlation coefficient of 0.86. Hence, when integrating various information provided by different ^13^C probes, HP ^13^C MRI may be able to assess the treatment efficacy of HCC and identify beneficiary patient cohort for specific treatments and even aid the development of novel therapeutic concepts.

Given the close relationship between pharmacologic action of chemotherapy and metabolic pathways, it is possible to utilize HP ^13^C MRI to evaluate chemotherapeutic response of liver malignancy. A recent pilot study investigated the dynamic metabolism in a prostate cancer patient with liver metastasis before and after chemotherapy.^15^ As shown in their research, the pyruvate‐to‐lactate conversion rate of the metastatic liver lesion decreased from 0.026 to 0.015 s^−1^ (42% reduction), and the tumour size reduced from 19.3 mm to 11.8 mm (39% reduction) 2 months after the initiation of chemotherapy. In addition, Cabella et al.^67^ proposed a novel preparation method for HP [5‐^13^C_1_] glutamine and confirmed its ability in measuring the response to chemotherapy in HCC cells. In light of these findings, HP ^13^C MRI may become the solution to current unmet clinical dilemma of liver cancer, which can be especially helpful in guiding the development of new chemotherapeutic and targeted drugs, and acting as a robust biomarker in longitudinal follow‐up trails.

## 5 FUTURE OPPORTUNITIES

HP ^13^C MRI provides a fundamentally new way of looking at patients with liver diseases. By identifying intracellular metabolism, we can expect to better understand the biology of diffuse liver diseases and therefore better manage them. These specific metabolic markers can be the non‐invasive surrogate in monitoring the disease progression and assessing therapeutic response. As for liver malignancies, the information on downstream metabolism offers the possibility of detecting early HCC before observable morphological changes and understanding its pathogenic mechanism. Such metabolic biomarkers can also help assess biological behaviours of tumours and aid personalized treatment strategies. For example, the metabolic alterations in tumour and peritumoral parenchyma may guide surgeons to determine the actual resected areas. In addition, based on the previous findings, HP ^13^C MRI can be a powerful tool in evaluating the treatment efficacy and predicting the clinical outcomes of patients. Despite the encouraging results and great potential of this technique in both diffuse and focal liver diseases, further research is still warranted to optimize the workflow.

Continuing works on the imaging hardware and acquisition sequences are needed to improve the sensitivity of HP ^13^C MRI and increase its clinical applicability. Besides the development and technical improvements of dual‐tuned ^1^H/^13^C volume coils for abdominal organs, works on receive surface coils with short coil‐to‐sample distance and optimal coil combinations are currently ongoing.^68^, ^69^ Additionally, in order to further prevent signal loss and capture rapid metabolic conversion without significant motion artefacts, new fast acquisition is required to obtain volumetric and dynamic HP MR data of the liver, preferably towards whole liver coverage, sub‐centimetre spatial resolution and higher temporal resolution (e.g. 1 s for pyruvate and 3 s for its metabolites). With the combination of parallel imaging technique, compress sensing and spiral trajectories,^70^ it is believed that current acquisition methods will advance to these expectations in the near future. More recently, a novel post‐processing pipeline was proposed to reduce background noise in HP spectra and increase the accuracy of kinetic estimations in patients with liver tumours,^71^ emphasizing the needs for new analysis techniques with improved spectral SNR and more accurate measurements.

In view of the heterogeneous nature of liver diseases, especially liver malignancies, the combination of HP ^13^C MRI with multiphasic and multiparametric ^1^H imaging, as well as multimodal findings could be of great significance for personalized medicine in the future. For example, the liver‐specific gadolinium contrast‐agent can not only provide valuable multiphasic information about haemodynamic changes but also selectively suppress normal hepatocyte contributions to hyperpolarized ^13^C MRI signal, which could be applied to separate the metabolic signals arising from the small tumours and surrounding normal hepatocytes.^72^ On the other hand, a multiparametric imaging approach with both HP ^13^C MRI and diffusional or other functional sequences is anticipated to monitor the therapeutic effects in liver diseases.^10^, ^29^, ^73^ Besides, the combination of HP ^13^C MRI and PET was shown promise in a rat model with HCC.^48^ This multimodal technique was named as HyperPET,^74^ and it is believed to provide complementary information and allow for better evaluation of the biological behaviours and treatment response in liver cancers.

In addition to the widely investigated pyruvate, a number of different ^13^C substrates have been proposed and tested promising in the liver, including earlier mentioned DHA, EAA, glutamine, urea and fumarate, as well as dihydroxyacetone,^75^, ^76^ alanine^77^ and several others^78^, ^79^ (Table 3), providing further details on the metabolism under different hepatic pathologies. As reported, dihydroxyacetone is sensitive to dysregulation of hepatic gluconeogenesis and glycolysis^75^, ^76^ and may be used to investigate metabolic diseases such as NAFLD or cirrhosis in a non‐invasive manner. Furthermore, alanine has a potential role in measuring hepatic redox reaction, which could be utilized in the evaluation of liver damage.^77^ Moreover, simultaneous co‐polarization and co‐injection of multiple compounds was recently shown feasible in a preclinical HCC model.^14^ This is significant because it enables the assessment of several cellular processes at the same time and optimizes the workflow and could possibly become a future research direction. Further work with higher and faster polarizations and simplified delivery of sterile HP probes are also needed to facilitate the clinical adoption of this technique.

**TABLE 3:** Established and promising hyperpolarized ^13^C probes in liver diseases

Molecule	Pathways / functions	Possible applications	References
[1‐^13^C] pyruvate	Glycolysis and ALT, LDH, PDH activity	Diffuse liver diseases and liver cancers	10, 11, 12, 13, 14, 15, 24, 25, 29, 34, 35, 46, 48, 49, 50, 56
[1‐^13^C] dehydroascorbic acid	Redox status	Liver injury and NASH	40
[1,3‐^13^C_2_] ethyl acetoacetate	Carboxylesterase activity	Liver cancers	51
[5‐^13^C] glutamine	Glutaminolysis, cellular proliferation	Liver cancers	58, 67
[^13^C] urea or [^13^C, ^15^N_2_] urea	Perfusion	Liver fibrosis and liver cancers	14, 59
[1,4‐^13^C_2_] fumarate	Cellular necrosis	Liver cancers	14
[2‐^13^C] dihydroxyacetone	Gluconeogenesis	NAFLD	75, 76
[1‐^13^C] alanine	Redox status	Liver injury and NASH	77
[1‐^13^C, U‐^2^H_5_] ethanol	Aldehyde dehydrogenase activity	Alcoholic liver disease and liver cancers	78
[1‐^13^C]‐_L_‐lactate	Pyruvate carboxylase activity	NAFLD	79

Abbreviations: ALT, alanine transaminase; LDH, lactate dehydrogenase; NAFLD, non‐alcoholic fatty liver disease; NASH, non‐alcoholic steatohepatitis; PDH, pyruvate dehydrogenase.

Last but not least, extended preclinical studies of liver metabolism are still needed to bridge the translational gap of HP ^13^C MRI. Currently, there is an ongoing clinical trial aiming to explore the effect of nutritional state and fatty liver on TCA cycle in healthy subjects and patients with fatty liver diseases by using HP ^13^C pyruvate injection.^80^ Nevertheless, owing to the limited human studies in real clinical scenarios, multicentre trails are necessary to better standardize this technique and improve its robustness, reliability and efficiency.^7^ Hopefully, with the help of multiple collaborations, HP ^13^C MRI can be integrated as a short (5‐min) add‐on into the routine MRI examination in the coming future.

## 6 CONCLUSIONS

HP ^13^C MRI is an emerging and promising technique in both diffuse liver diseases and liver malignancies. It allows for real‐time visualization of hepatic metabolism and enzymatic conversions and is of significant potential in detecting liver diseases, monitoring its progression and assessing the treatment efficacy. There are still unresolved barriers of this technique on the road to clinical transformation, but it also brings future research opportunities in this field. With the improvement of this technique and optimization of the workflow, HP ^13^C MRI is expected to become a powerful tool for the diagnosis and management of liver diseases in clinical practice.

## CONFLICT OF INTEREST

All authors declare no conflict of interest.

## Data Availability

Data sharing is not applicable to this article as no new data were created or analysed in this study.
